# Relationships among tumor necrosis factor‐alpha levels, beta‐amyloid accumulation, and hippocampal atrophy in patients with late‐life major depressive disorder

**DOI:** 10.1002/brb3.70016

**Published:** 2024-09-05

**Authors:** Szu‐Kai Ho, Ing‐Tsung Hsiao, Kun‐Ju Lin, Yi‐Ming Wu, Kuan‐Yi Wu

**Affiliations:** ^1^ Department of Psychiatry Chang Gung Memorial Hospital and College of Medicine Chang Gung University Taoyuan Taiwan; ^2^ Department of Nuclear Medicine and Center for Advanced Molecular Imaging and Translation Chang Gung Memorial Hospital Taoyuan Taiwan; ^3^ Department of Medical Imaging and Radiological Sciences and Healthy Aging Research Center Chang Gung University Taoyuan Taiwan; ^4^ Neuroscience Research Center Chang Gung Memorial Hospital, Linkou Medical Center Taoyuan Taiwan; ^5^ Department of Radiology Chang Gung Memorial Hospital Taoyuan Taiwan

**Keywords:** 18F‐florbetapir (AV‐45/Amyvid), amyloid, hippocampal atrophy, major depressive disorder, TNF‐α

## Abstract

**Background:**

Major depressive disorder (MDD) is characterized by hippocampal volume reduction, impacting cognitive function. Inflammation, particularly elevated tumor necrosis factor‐alpha (TNF‐α) levels, is consistently implicated in MDD pathophysiology. This study investigates the relationships between TNF‐α levels, hippocampal volume, beta‐amyloid (Aβ) burden, and cognitive abilities in MDD patients, aiming to illuminate the complex interplay among inflammatory markers, pathology indicators, structural brain alterations, and cognitive performance in non‐demented MDD individuals.

**Method:**

Fifty‐two non‐demented MDD patients, comprising 25 with mild cognitive impairment (MCI), were recruited along with 10 control subjects. Each participant underwent a thorough assessment encompassing TNF‐α blood testing, ^18^F‐florbetapir positron emission tomography, magnetic resonance imaging scans, and neuropsychological testing. Statistical analyses, adjusted for age and education, were performed to investigate the associations between TNF‐α levels, adjusted hippocampal volume (HVa), global Aβ burden, and cognitive performance.

**Results:**

MCI MDD patients displayed elevated TNF‐α levels and reduced HVa relative to controls. Correlation analyses demonstrated inverse relationships between TNF‐α level and HVa in MCI MDD, all MDD, and all subjects groups. Both TNF‐α level and HVa exhibited significant correlations with processing speed across all MDD and all subjects. Notably, global ^18^F‐florbetapir standardized uptake value ratio did not exhibit significant correlations with TNF‐α level, HVa, and cognitive measures.

**Conclusion:**

This study highlights elevated TNF‐α levels and reduced hippocampal volume in MCI MDD patients, indicating a potential association between peripheral inflammation and structural brain alterations in depression. Furthermore, our results suggest that certain cases of MDD may be affected by non‐amyloid‐mediated process, which impacts their TNF‐α and hippocampal volume. These findings emphasize the importance of further investigating the complex interplay among inflammation, neurodegeneration, and cognitive function in MDD.

## Graphical abstract

Patients with MDD and MCI exhibited higher TNF‐α levels and reduced hippocampal volumes compared to controls. An inverse relationship was observed between TNF‐α levels and hippocampal volume in the MCI MDD group, the overall MDD group, and across all subjects. No significant correlations were found between beta‐amyloid deposition and TNF‐α levels, hippocampal volume, or cognitive measures.

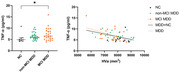

## 1 INTRODUCTION

Major depressive disorder (MDD) is a complex neuropsychiatric condition with far‐reaching implications for both mental and physical health. Existing neuroimaging research consistently indicates a diminished hippocampal volume in individuals with unipolar depression (Hickie et al., 2005). Hippocampal volume has been linked to domains of cognition such as processing speed, working memory, spatial navigation, and abstract reasoning (Hardcastle et al., 2020). In our prior study (Wu et al., 2018), we also observed substantial hippocampal atrophy in a considerable portion of individuals experiencing late‐life major depression and mild cognitive impairment (MCI). Despite this finding, the precise mechanism underlying reduced hippocampal volume remains elusive. Nevertheless, prevailing consensus attributes this phenomenon to glucocorticoids resulting from dysregulation of the hypothalamic‐pituitary‐adrenal (HPA) axis, potentially contributing to both hippocampal atrophy and functional impairments (Naismith et al., 2012). The dysregulation of the HPA axis results in the overactivation of the immune system, leading to the excessive release of inflammatory molecules and tilting the balance toward a pro‐inflammatory state (Hassamal, 2023).

There is common agreement that inflammatory processes play a critical role in the pathophysiology of MDD (Frodl & Amico, 2014). Research indicates that individuals with depression exhibit heightened levels of C‐reactive protein, pro‐inflammatory cytokines such as interleukin (IL)‐1, IL‐6, and tumor necrosis factor‐alpha (TNF‐α), along with chemokines (Dowlati et al., 2010). Notably, TNF‐α, among these cytokines, consistently emerges in the literature as significantly linked to the pathophysiology of MDD (Bortolato et al., 2015). Aberrations in TNF‐α levels have been demonstrated to impact the severity of psychiatric symptoms and treatment response (Uzzan & Azab, 2021). Furthermore, TNF‐α antagonists have shown antidepressant and pro‐cognitive effects in mouse models (Belarbi et al., 2012), providing additional insights into the potential therapeutic relevance of targeting inflammatory pathways in the context of depression.

Some magnetic resonance imaging (MRI) research suggests that neuroinflammation in MDD could be linked to structural and functional abnormalities across various adjusts of the central nervous system, including the hippocampus (Frodl et al., 2012); however, the definitive establishment of a causal relationship between inflammation and these changes remains a subject of ongoing exploration (Frodl & Amico, 2014). In addition, Alzheimer's disease (AD), characterized by amyloid plaques and neurofibrillary tangles, is associated with neuroinflammation. Beta‐amyloid (Aβ) deposition is postulated to initiate a cascade of cerebral neuroinflammation, leading to an increase in pro‐inflammatory cytokines such as TNF‐α (Wang et al., 2015). In the present study, we investigated the association between TNF‐α level, hippocampal volume, Aβ burden, and cognitive performance in MDD patients. These findings aim to contribute to unraveling the complex connections among inflammatory markers, brain Aβ pathology, brain structural volumes, and cognitive function in MDD patients.

## 2 METHODS

### 2.1 Subjects

This study constitutes a comprehensive cross‐sectional and prospective investigation carried out at the Geriatric Psychiatry Center of Chang Gung Memorial Hospital from October 2014 to June 2017. Individuals were diagnosed with MDD by assessing lifetime major depressive episodes, age at onset, episode frequency, and late‐onset MDD (defined as onset after age 60). Assessment methods included clinical structured interviews and retrospective medical chart reviews based on the Diagnostic and Statistical Manual of Mental Disorders (DSM)‐IV [or DSM‐5 (American Psychiatric Association, 2013) after 2016] criteria. Control subjects consisted of cognitively normal individuals (mini mental status examination [MMSE] ≥ 27 and clinical dementia rating [CDR] = 0) without any history of psychiatric disorders. Inclusion criteria specified participants aged 50 years or older, free from neurologic conditions affecting brain structure (e.g., stroke, traumatic head injury, and epilepsy), stable medical conditions involving vital organs (heart, lungs, liver, kidneys), and no recent history of alcohol or substance abuse within the past year. Additionally, participants were screened to ensure that they did not meet the criteria for dementia associated with AD, as per the National Institute on Aging and Alzheimer's Association (NIA‐AA) criteria (Jack et al., 2018), International Working Group (IWG) criteria for typical/atypical AD dementia (Dubois et al., 2014), or DSM‐5 criteria for any type of dementia (American Psychiatric Association, 2013).

Eligible cases were conditioned on completion of ^18^F‐florbetapir positron emission tomography (PET)/MR scan, brain MRI and TNF‐α blood test, and neuropsychological testing. TNF‐α was measured by chemiluminescent immunoassay using IMMULITE/IMMULITE 1000 (SIEMENS). The assay sensitivity for TNF‐α was 1.7 pg/mL. Apolipoprotein E (APOE) genotypes were determined by polymerase chain reaction study. Comprehensive neuropsychological tests were conducted on all participants as per our previous study (Wu et al., 2016). A total of 52 MDD patients and 10 non‐depressed control subjects were included in the study. Written informed consent was obtained from all participants, and the study protocol was approved by the Institutional Review Boards of the Ministry of Health and Welfare and Chang Gung Medical Center.

### 2.2 Neuropsychological tests and MCI among MDD patients

Cognitive assessment in this study was conducted following the methods outlined in a previous study (Wu et al., 2016). The evaluation included a global screening with the MMSE and the CDR, along with domain‐specific assessments utilizing a comprehensive battery of neuropsychological tests. These tests served to validate the cognitive normalcy of control participants and to classify MDD patients into MCI and non‐MCI subgroups.

The neuropsychological battery comprised various tests: the Wechsler Adult Intelligence Scale‐Third Edition (WAIS‐III) digit symbol and Trail‐making A tests for evaluating information‐processing speed, the Controlled Oral Word Association, Frontal Assessment Battery, Trail‐making B, and WAIS‐III‐similarity tests for assessing executive function, the 12‐item, six‐trial selective reminding test for memory evaluation, the WAIS‐III‐language test for language assessment, and the WAIS‐III‐digit span test for attention assessment.

Scores were standardized into *z*‐scores using regression‐based norms, adjusted for age and educational level with reference to independent normative data for Taiwan. MCI was determined in MDD patients demonstrating impairment in at least one cognitive domain, indicated by a score of 1.5 standard deviations (SDs) below the age‐ and education‐adjusted norm. MDD subjects had a CDR score of 0 or 0.5.

### 2.3 MRI image acquisition and analysis

T1‐weighted MRI scans were conducted on all subjects utilizing a 3T Siemens Magnetom TIM Trio scanner on PET/MR (Siemens Medical Solutions). The acquisition employed a sagittal T1‐weighted magnetization prepared rapid acquisition gradient echo sequence with the following parameters: repetition time/echo time = 2600/3.12 ms, inversion time = 900 ms, flip angle = 13°, and voxel size = 0.5 mm × 0.5 mm × 1.1 mm. FreeSurfer version 5.3 image analysis software was utilized for processing structural scans to determine total bilateral hippocampal and intracranial volumes (Dale et al., 1999). A linear‐regression normalization method was applied (Voevodskaya et al., 2014), with the total bilateral hippocampal volume adjusted by the estimated total intracranial volume to obtain the adjusted hippocampal volume (HVa), as described in our previous study (Wu et al., 2018), to reduce inter‐subject variability.

### 2.4 Amyloid PET acquisition and analysis

Radiosynthesis of ^18^F‐florbetapir (Yao et al., 2010) and amyloid PET data acquisition followed the same procedures as previously described (Lin et al., 2010). During the study, each ^18^F‐florbetapir PET scan (380 ± 5 MBq) at 50–60 min postinjection was obtained using a Biograph mMR PET/MR System (Siemens Medical Solutions). The 3D ordered subsets expectation‐maximization PET reconstruction algorithm (3 iterations, 21 subsets; Gaussian filter: 2 mm; zoom: 3) with MR‐based attenuation correction, scatter, and random corrections was applied to obtain PET images with a matrix size of 344 × 344 × 127 and a voxel size of 0.83 mm^3^ × 0.83 mm^3^ × 2.03 mm^3^.

All image data were processed and analyzed using PMOD image analysis software (version 3.3; PMOD Technologies Ltd). Each PET image was coregistered to the corresponding T1‐weighted MR image, and the individual T1‐weighted MR images were spatially normalized to the Montreal Neurological Institute MRI template. The whole cerebellum was used as the reference region for calculating the standardized uptake value ratios (SUVR) to obtain the final normalized global cortical SUVR for each subject.

### 2.5 Statistical analysis

Descriptive statistics were presented as means ± SD or absolute numbers with proportions for demographic and clinical data. Nonparametric statistical methods, specifically the Kruskal–Wallis test with Dunn's post hoc multiple comparisons for three‐group analyses and the Mann–Whitney *U* test for two‐group comparisons, were employed to examine differences in continuous variables. For categorical data, the *χ* ^2^ test was utilized, with Fisher's exact test applied for APOE4 due to small sample sizes. Partial correlations between TNF‐α, HVa, and global ^18^F‐florbetapir SUVR across sample subgroups were assessed using Pearson correlation analysis adjusted for age and years of education. Additionally, Pearson correlation analysis adjusted for age and years of education was used to evaluate partial correlations between TNF‐α, HVa, global ^18^F‐florbetapir SUVR, neuropsychological test data, and depression characteristics. Statistical analysis was performed using IBM SPSS version 25.0 (IBM Corp.), with statistical significance set at *p* < .05.

## 3 RESULTS

The study recruited 52 non‐demented MDD patients and 10 control subjects. Among the MDD patients, 25 (48.1%) met the definition of MCI. The demographic and clinical characteristics of each group are shown in Table 1. The groups did not differ significantly in age, education, and APOE4 carriers. The non‐MCI MDD and MCI MDD patients had similar Hamilton Depression Rating Scale (HAM‐D) score, in addition to similar clinical depressive features (age at onset, disease duration, and number of depressive episodes).

**TABLE 1:** Demographic and clinical characteristics of the non‐mild cognitive impairment major depressive disorder (MCI MDD), MCI MDD, and the control subjects.

Characteristic	non‐MCI MDD	MCI MDD	Controls	*p* Value
	*n* = 27	*n* = 25	*n* = 10	
Age (years)				
Mean ± SD	62.1 ± 6.3	63.8 ± 5.8	59.5 ± 2.9	.072
Female gender, *n* (%)	25 (92.6)	21 (84.0)	5 (50.0)	.013
Education (years)				
Mean ± SD	8.6 ± 3.4	7.8 ± 4.7	11.4 ± 4.5	.104
HAM‐D				
Mean ± SD	9.0 ± 5.3**^a^	12.5 ± 6.2***^a^	2.1 ± 2.2	<.001
MMSE				
Mean ± SD	27.2 ± 2.3	23.9 ± 3.7***^a,^ **^b^	28.6 ± 1.3	<.001
CDR 0.5, *n* (%)	8 (29.6)***^c^	22 (88.0)***^c^	0	<.001
APOE4, *n* (%)	4 (14.8)	6 (24.0)	0	.273
Age at onset (years)				
Mean ± SD	50.0 ± 11.7	54.3 ± 8.1	–	.113
Duration since onset of depression (years)				
Mean ± SD	11.6 ± 10.3	10.9 ± 9.8	–	.698
Number of depressive episodes				
Mean ± SD	2.0 ± 1.3	2.2 ± 1.3	–	.801

*Note*: *p* Values denote the significance of differences among the non‐MCI MDD, MCI MDD, and control groups using the Kruskal–Wallis test (continuous variables) or the *χ* ^2^ test (categorical variables).

Abbreviations: APOE4, Apolipoprotein E *ε*4 carrier; CDR, clinical dementia rating; HAM‐D, 17‐item Hamilton Depression Rating Scale; MMSE, mini mental status examination.

^a^ Dunn's post hoc analysis, significant difference between the non‐MCI MDD or MCI MDD group and the control subjects; *p < .05, **p < .01, and ***p < .001.

^b^ Dunn's post hoc analysis, significant difference between the non‐MCI MDD patients and the MCI MDD patients; *p < .05, **p < .01, and ***p < .001.

^c^ Mann–Whitney *U* test or *χ* ^2^ test (Fisher's exact test for APOE4), significant difference between each MDD group and the control subjects; *p < .05, **p < .01, and ***p < .001.

**FIGURE 1:**
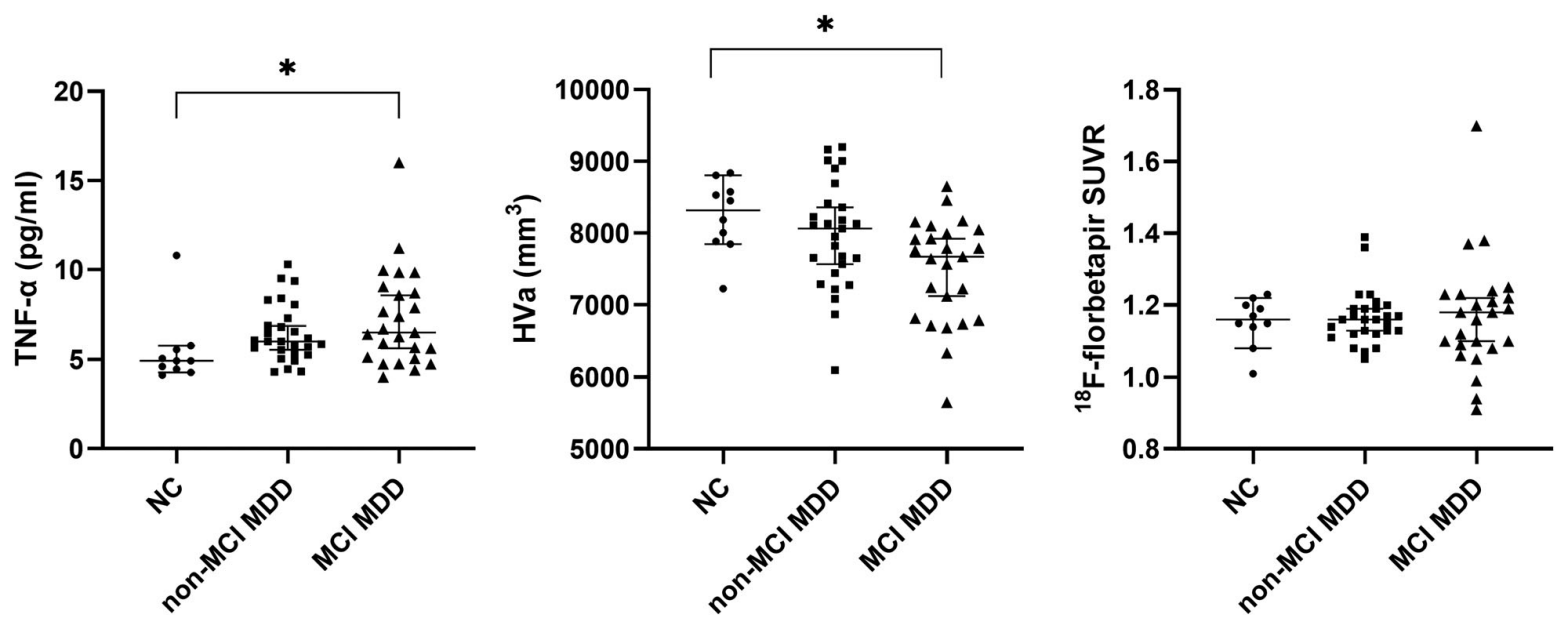
**Biomarker distributions**. This shows tumor necrosis factor‐alpha (TNF‐α), adjusted hippocampal volume (HVa), and global ^18^F‐florbetapir standardized uptake value ratios (SUVR) distributions in the controls (*n* = 10), non‐mild cognitive impairment major depressive disorder (MCI MDD) (*n* = 27), and the MCI MDD (*n* = 25) subjects. *p < .05 using the Kruskal–Wallis test with Dunn's post hoc analysis. NC, normal control.

Comparing the non‐MCI MDD, MCI MDD, and control groups, Table 2 shows significant differences in TNF‐α level (*p* = .026) and HVa (*p* = .008), but no difference in the global ^18^F‐florbetapir SUVR. The post hoc analysis showed that the MCI MDD group exhibited significantly higher TNF‐α level and lower HVa compared with the controls. The biomarker distributions are displayed in Figure 1. Compared with the controls, the whole 52 MDD patients also showed significantly increased TNF‐α level and decreased HVa (Figure S1).

**TABLE 2:** Tumor necrosis factor‐alpha (TNF‐α) levels, adjusted hippocampal volume (HVa), global ^18^F‐florbetapir standardized uptake value ratios (SUVR), and cognitive characteristics of the non‐mild cognitive impairment major depressive disorder (MCI MDD), MCI MDD, and the control subjects.

Characteristic	non‐MCI MDD	MCI MDD	Controls	*p* Value
	*n* = 27	*n* = 25	*n* = 10	
TNF‐α				
Mean ± SD	6.5 ± 1.6	7.3 ± 2.7*^a^	5.5 ± 2.0	.026
HVa				
Mean ± SD	7970.9 ± 754.4	7478.9 ± 723.7*^a^	8235.6 ± 504.2	.008
Global ^18^F‐florbetapir SUVR				
Mean ± SD	1.2 ± .1	1.2 ± .2	1.2 ± .1	.995
Cognitive domain *z*‐scores, Mean ± SD				
Executive function	−.1 ± .6	−.7 ± .6**^a^ ^,^ **^b^	.2 ± .4	<.001
Memory	−.3 ± .7	−1.2 ± .9**^a^ ^,^ **^b^	.0 ± .7	<.001
Processing speed	−.5 ± .6	−1.7 ± .8***^a^ ^,^ ***^b^	.3 ± .9	<.001
Visuospatial function	.2 ± .7	−.2 ± 1.1	.4 ± .6	.121
Language	1.2 ± .8	.6 ± .7*^a^ ^,^ *^b^	1.5 ± 1.0	.006
Attention	.4 ± .8	−.5 ± .9*^a^ ^,^ **^b^	.5 ± 1.1	.001

*Note*: *p* Values denote the significance of differences among the non‐MCI MDD, MCI MDD, and control groups using the Kruskal–Wallis test (continuous variables) or the *χ* ^2^ test (categorical variables).

^a^ Dunn's post hoc analysis, significant difference between the non‐MCI MDD or MCI MDD group and the control subjects; *p < .05, **p < .01, and ***p < .001.

^b^ Dunn's post hoc analysis, significant difference between the non‐MCI MDD patients and the MCI MDD patients; *p < .05, **p < .01, and ***p < .001.

In terms of neuropsychological testing, the MCI MDD patients had greater cognitive deficits than the control subjects in all neuropsychological tests except for visuospatial function; the most severe deficits occurred in executive function (*p* < .001), memory (*p* < .001), and processing speed domains (*p* < .001). No differences in every domain of cognition were found between non‐MCI MDD group and control subjects after post hoc analyses.

**FIGURE 2:**
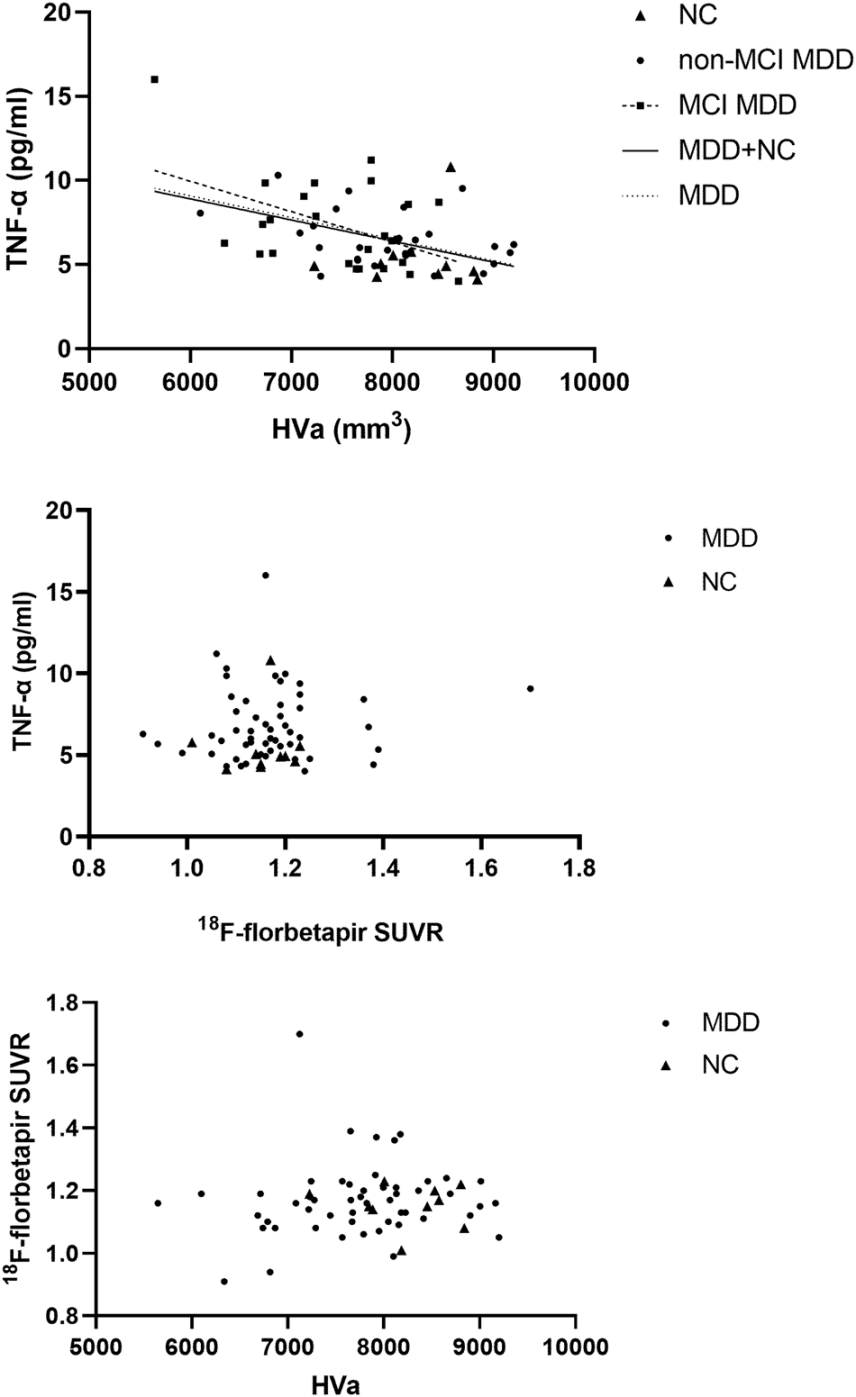
**Relationships among** tumor necrosis factor‐alpha (TNF‐α), adjusted hippocampal volume (HVa)**, and global ^18^F‐florbetapir** standardized uptake value ratios (SUVR) in **the different sample groups**. Significant negative correlations between TNF‐α level and HVa were observed in mild cognitive impairment major depressive disorder (MCI MDD) (*r* = −.452, *p* = .030), all MDD (*r* = −.394, *p* = .005) and all subjects (*r* = −.369, *p* = .004) groups. NC, normal control.

To assess the relationship among TNF‐α level, HVa, and Aβ burden, separate partial correlation analyses were conducted in the MCI MDD, non‐MCI MDD, all MDD, and all subjects groups. After controlling for age and level of education, significant negative correlations between TNF‐α level and HVa were observed in MCI MDD (*r* = −.452, *p* = .030), all MDD (*r* = −.394, *p* = .005), and all subjects (*r* = −.369, *p* = .004) groups (Figure 2). However, there were no correlations either between TNF‐α level and ^18^F‐florbetapir SUVR, or between ^18^F‐florbetapir SUVR and HVa in each group.

**FIGURE 3:**
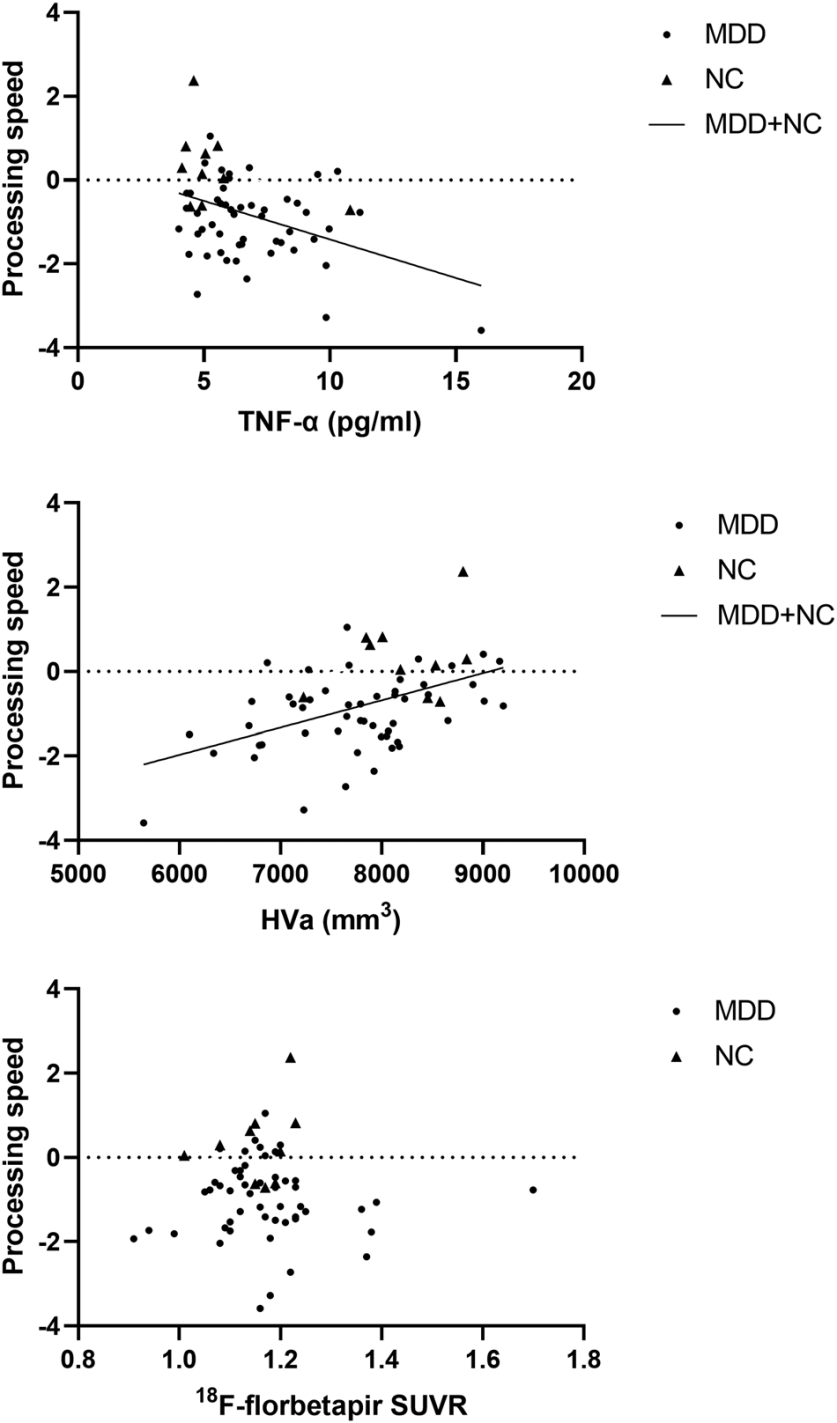
**Correlations of the processing speed function with** tumor necrosis factor‐alpha **(TNF‐α),** adjusted hippocampal volume **(HVa), and global ^18^F‐florbetapir** standardized uptake value ratios **(SUVR) in the different sample groups**. TNF‐α level and HVa were both significantly correlated with processing speed function when all subjects were included. MDD, major depressive disorder; NC, normal control.

We calculated the correlations between the above biomarkers and HAM‐D score, MMSE score, as well as each neuropsychological test score across different sample groups. Figure 3 shows that TNF‐α level and HVa were both significantly correlated with processing speed when all subjects were included. HVa was significantly correlated with MMSE score in all subjects and all MDD groups. TNF‐α level was significantly negatively correlated with HAM‐D score only in MCI MDD patients. For each cognitive domain, HVa was significantly positively correlated with processing speed, language, memory, and attention in all subjects and all MDD groups. The ^18^F‐florbetapir SUVR was not significantly correlated with HAM‐D score, MMSE score, or each neuropsychological test score in different sample groups. Details of partial correlation coefficients are shown in Table 3.

**TABLE 3:** Correlations of cognitive functions with tumor necrosis factor‐alpha (TNF‐α), adjusted hippocampal volume (HVa), and global ^18^F‐florbetapir standardized uptake value ratios (SUVR) in all subjects and major depressive disorder (MDD) groups.

	TNF‐α		HVa		^18^F‐florbetapir SUVR	
	MDD and controls	MDD	MDD and controls	MDD	MDD and controls	MDD
	*n* = 62	*n* = 52	*n* = 62	*n* = 52	*n* = 62	*n* = 52
MMSE	−.263*	−.234	.260*	.290*	−.150	−.156
Executive function	−.229	−.165	.226	.139	−.036	−.034
Memory	−.195	−.156	.448***	.408**	−.059	−.081
Processing speed	−.372**	−.304*	.445***	.437**	.005	−.003
Visuospatial function	−.231	−.204	.230	.193	.162	.174
Language	−.228	−.184	.322*	.318*	−.089	−.114
Attention	−.163	−.070	.430***	.382**	.121	.152

Abbreviation: MMSE, mini mental status examination.

*p < .05, **p < .01, and ***p < .001 after adjustment for age and years of education.

## 4 DISCUSSION

Our results suggest that MCI MDD patients had higher levels of TNF‐α and reduced hippocampal volume compared to controls. There was a significant negative correlation between TNF‐α levels and HVa in the MCI MDD, all MDD, and all subjects groups. However, there were no significant differences in Aβ burden between the groups. No significant correlations were found between global Aβ burden and TNF‐α level, and hippocampal atrophy. TNF‐α level and HVa showed significant correlations with processing speed across all MDD and all subjects, but the global Aβ burden did not show significant correlations with cognitive performance in different sample groups.

The study reveals a negative correlation between TNF‐α levels and hippocampal volume, supporting the association of peripheral inflammation markers with structural anomalies, aligning with prior research in patients with MDD (Bai et al., 2020). In substantial patients, prolonged exposure to stress serves as an underlying risk factor for the development of neuroinflammation and depression (Hassamal, 2023). The dysregulation of the HPA axis and the activation of immune response patterns explain the primary mechanism connecting chronic stress, inflammation, and depression (Fasick et al., 2015). Continuous exposure to stress leads to an overstimulation of the HPA axis, leading to an excessive release of cortisol. This, in turn, causes a dysregulation of the glucocorticoid negative feedback loop, as a result of glucocorticoid receptor resistance. Ultimately, this resistance contributes to the release of pro‐inflammatory cytokines, including TNF‐α, IL‐1, and IL‐6 (Hassamal, 2023; Mikulska et al., 2021). Conversely, the induction of TNF‐α triggers the activation of the HPA axis, enhancing anti‐inflammatory glucocorticoids but diminishing their responsiveness. The reduced glucocorticoid‐responsiveness leads to a surge in pro‐inflammatory cytokines that impede glucocorticoid‐mediated suppression, ultimately resulting in a vicious cycle (Fasick et al., 2015). Elevated levels of glucocorticoids have been linked to hippocampal atrophy. This mechanism includes the hippocampal dendritic atrophy, attenuation of hippocampal neurogenesis, and an increased risk to cell death (Campos et al., 2020; Sudheimer et al., 2014). Concerning pro‐inflammatory cytokines such as TNF‐α, some studies propose that they may influence hippocampal atrophy by inducing systemic hypercortisolemia; other research suggests that they can directly impact neuronal death by degrading the blood–brain barrier (Sudheimer et al., 2014). Regardless, our research findings suggest that the elevated pro‐inflammatory cytokine is associated with structural alterations in the hippocampus.

In the present study, no significant correlations were found between Aβ burden and HVa in any of the groups. The findings are in line with previous findings in which the association between amyloid positivity and hippocampal volume loss was nonsignificant in cognitively intact elderly cases (Haller et al., 2019). Another study findings revealed that hippocampal volume was not significantly associated with amyloid status in the healthy control, early MCI, or AD dementia group (Trzepacz et al., 2016). In a study conducted by Jack et al. (2015) involving 1209 cognitively healthy individuals aged 50–95 years, it was found that HVa reduction occurs at earlier ages compared to the presence of abnormal amyloid PET. Our previous research (Wu et al., 2018) also revealed that a substantial proportion of patients experiencing late‐life major depression and MCI showed signs of hippocampal atrophy, despite testing negative for brain Aβ accumulation. Historically, our focus in accurately diagnosing AD has predominantly centered around amyloid positivity. Nevertheless, there is a growing exploration into the significance of subthreshold levels of Aβ (Bischof & Jacobs, 2019). Non‐amyloid neurodegeneration may link to subthreshold Aβ changes (Jack et al., 2013). These suspected non‐Alzheimer pathophysiology MDD patients provide insight into the different neuropathologies (Wu et al., 2023). For example, hippocampal sclerosis, acknowledged as a component of limbic‐predominant age‐related TDP‐43 encephalopathy, could be considered among the potential contributing conditions (Nelson et al., 2019). Recent studies have described more profound hippocampal atrophy in subjects with hippocampal sclerosis than in those with other pathologies such as AD neuropathologic change (Yu et al., 2020). In our study, the distribution of Aβ levels among MCI MDD patients was broad (Figure 1), suggesting potential inclusion of diverse subgroups exhibiting AD or non‐AD pathologies. This might explain the lack of significant correlations found between Aβ burden and HVa. Moreover, there was no significant difference in amyloid pathology burden compared to the healthy control group. This result also highlights the considerable heterogeneity in neurodegenerative pathophysiology in depression in old age.

We observed that HVa was positively associated with several neuropsychological tests in all MDD and all subjects groups. This was in accordance with earlier studies that have shown smaller hippocampal volumes in patients with MCI, compared with healthy controls (Fox et al., 1996). Haller et al. (2019) also demonstrated that progressive decrement of neuropsychological performance is associated with hippocampal volume loss in asymptomatic subjects. Although our study did not find a correlation between Aβ burden and neuropsychological test results, we did not refute the possible association between Aβ accumulation and cognitive decline. Several reports have found significant inverse correlations between Aβ burden and memory dysfunction, as well as a longitudinal decline in cognition (Hanseeuw et al., 2019; Wu et al., 2016). Apart from the possibility that the sample size in this study might not have been large enough to be representative, the main reason is that the wide dispersion of Aβ levels among MDD cases in this study could obscure the correlation analysis between Aβ accumulation and cognitive decline.

Our findings demonstrated that TNF‐α level was negatively correlated with some neuropsychological tests, especially processing speed, in all MDD and all subjects groups. Recent research has made notable advancements in understanding how certain cytokines within the brain influence processes such as learning, memory, and plasticity. TNF‐α is a versatile cytokine that significantly impacts various cognitive functions within the brain, playing a substantial role in both physiological and pathological conditions (Raffaele et al., 2020). Previous studies have shown that the TNF‐α levels in the peripheral blood of AD group were higher than in that of healthy control group (Swardfager et al., 2010), and that elevated levels of TNF‐α from the periphery, which are linked to systemic inflammation, are associated with cognitive decline in AD patients (Kim et al., 2017). Moreover, TNF‐α is associated with the harmful effects caused by Aβ in impairing learning and memory functions in AD (Wang et al., 2015). Although TNF‐α antagonism has shown promise in reducing depressive symptoms in chronic inflammatory diseases, there is currently a lack of direct studies exploring the impact of TNF‐α on cognitive impairment in MDD (Bortolato et al., 2015). Our study suggested a correlation between cognitive function and TNF‐α in patients with MDD. Additional research might be necessary to assess the intricate interplay between immune mechanisms and cognitive performance.

This study had several limitations. First, the study was limited to small sample sizes. Due to the limited sample size, it was not feasible to categorize MCI MDD patients into distinct subgroups based on varying domains of cognitive deficits, such as amnestic or non‐amnestic MCI. Second, the wide dispersion of Aβ levels in MDD in our study suggests potential inclusion of various subgroups comprising patients with AD or non‐AD pathologies (Figure 1 and Figure S1). Consequently, the analysis of correlations between Aβ, TNF‐α, HVa, and cognitive function may be limited. Third, cerebrospinal fluid (CSF) biomarkers were unavailable in this study. Therefore, we lack the capability to determine whether elevated biomarkers in the blood correspondingly increase in the CSF or elucidate their specific correlation. Fourth, due to the cross‐sectional nature of our study, we were unable to determine a cause‐and‐effect relationship between serum cytokine levels and cognitive dysfunction. Finally, interpretation and generalization of the findings to nondepressed subjects must acknowledge the potential limitations of this study. Future long‐term studies involving a larger number of participants and a wider array of neurodegenerative biomarkers are required to thoroughly explore the neurodegenerative mechanisms in elderly patients with depression.

## 5 CONCLUSION

Our study suggested that elevated peripheral TNF‐α levels in MCI MDD patients were associated with reduced hippocampal volume, highlighting a potential link between peripheral inflammation and structural anomalies in the brain. The absence of a correlation between Aβ and TNF‐α, and hippocampal atrophy in MDD may be linked to undisclosed factors related to non‐amyloid‐mediated processes. The observed correlations between TNF‐α, hippocampal volume, and cognitive performance underscore the complex relationship between inflammation and cognitive function in MDD.

## AUTHOR CONTRIBUTIONS

**Szu‐Kai Ho**: Conceptualization; formal analysis; validation; writing—original draft; writing—review and editing. **Ing‐Tsung Hsiao**: Data curation; formal analysis; investigation; resources; software; supervision. **Kun‐Ju Lin**: Conceptualization; investigation; methodology; resources; software; supervision. **Yi‐Ming Wu**: Data curation; investigation; resources; software. **Kuan‐Yi Wu**: Conceptualization; formal analysis; methodology; project administration; resources; supervision; validation; writing—original draft; writing—review and editing.

## CONFLICT OF INTEREST STATEMENT

The authors declare that they have no conflicts of interest.

### PEER REVIEW

The peer review history for this article is available at https://publons.com/publon/10.1002/brb3.70016.

## Supporting information


**Figure S1 Biomarker distributions in the control subjects and all MDD group**. This shows TNF‐α, HVa, and global ^18^F‐florbetapir SUVR distributions in the controls (*n* = 10) and the all MDD (*n* = 52) group. *p < .05, **p < .01 using the Mann–Whitney *U* test. HVa, adjusted hippocampal volume; MDD, major depressive disorder; NC, normal control; SUVR, standardized uptake value ratios; TNF‐α, tumor necrosis factor‐alpha.

## Data Availability

The raw data supporting the conclusions of this article will be made available by the authors, without undue reservation.
