# Supplementary figures and images for: Relationships among tumor necrosis factor‐alpha levels, beta‐amyloid accumulation, and hippocampal atrophy in patients with late‐life major depressive disorder
Source: Brain Behav. 2024 Sep 5;14(9):e70016. doi: 10.1002/brb3.70016 (PMC11376440; doi:10.1002/brb3.70016)

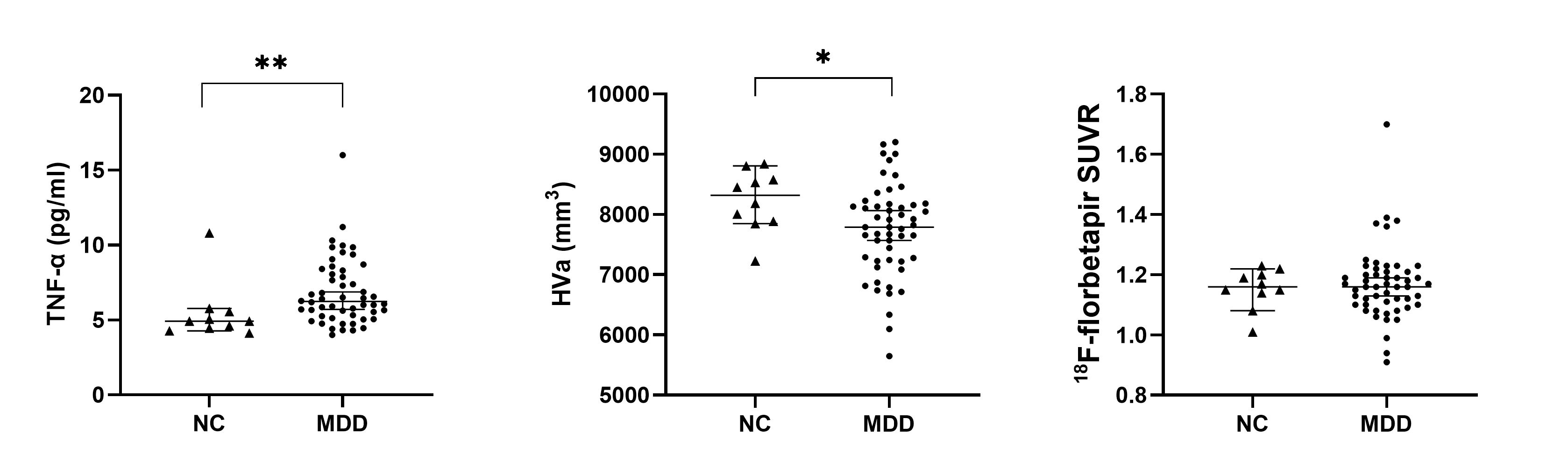

Supplement: Supplementary file 1 — Figure S1 Biomarker distributions in the control subjects and all MDD group. This shows TNF‐α, HVa, and global 18F‐florbetapir SUVR distributions in the controls (n = 10) and the all MDD (n = 52) group. *p < .05, **p < .01 using the Mann–Whitney U test. HVa, adjusted hippocampal volume; MDD, major depressive disorder; NC, normal control; SUVR, standardized uptake value ratios; TNF‐α, tumor necrosis factor‐alpha. [file BRB3-14-e70016-s001.tif]
